# Decision-making factors for an autologous stem cell transplant for older adults with newly diagnosed multiple myeloma: A qualitative analysis

**DOI:** 10.3389/fonc.2022.974038

**Published:** 2023-01-27

**Authors:** Owais Mian, Martine Puts, Arleigh McCurdy, Tanya M. Wildes, Mark A. Fiala, Matthew Kang, Mary Salib, Shabbir Alibhai, Hira Mian

**Affiliations:** ^1^ Department of Internal Medicine, University of Toronto, Toronto, ON, Canada; ^2^ Lawrence S. Bloomberg Faculty of Nursing, University of Toronto, Toronto, ON, Canada; ^3^ Department of Medicine, Division of Hematology, University of Ottawa, Ottawa, ON, Canada; ^4^ Division of Hematology and Oncology, Department of Internal Medicine, University of Nebraska Medical Center, Nebraska Medicine, Omaha, NE, United States; ^5^ College for Public Health and Social Justice, Saint Louis University, St Louis, MO, United States; ^6^ Department of Oncology, Washington University School of Medicine, St Louis, MO, United States; ^7^ Department of Oncology, Joseph Brant Hospital, Burlington, ON, Canada; ^8^ Department of Oncology, Walker Family Cancer Centre St. Catherine’s General Hospital, St. Catherine, ON, Canada; ^9^ Department of Medicine, University Health Network & University of Toronto, Toronto, ON, Canada; ^10^ Department of Oncology, McMaster University, Hamilton, ON, Canada

**Keywords:** multiple myeloma, autologous stem cell transplant (ASCT), treatment decision– making, qualitative, aged

## Abstract

**Purpose:**

A utologous stem cell transplant (ASCT) remains a standard of care among older adults (aged ≥65) with multiple myeloma (MM). However, heterogeneity in the eligibility and utilization of ASCT remains. We identified decision-making factors that influence ASCT eligibility and utilization among older adults with MM.

**Methods:**

A qualitative study across two academic and two community centres in Ontario was conducted between July 2019-July 2020. Older adults with MM (newly diagnosed MM aged 65-75 in whom a decision had been made about ASCT in <12 months) and treating oncologists completed a baseline survey and a subsequent interview, which was analyzed using thematic analysis.

**Results:**

Eighteen patients completed the survey and 9 follow-up interviews were conducted. Patients were happy with their treatment decision with “trust in their oncologist” and “wanting the best treatment” as the most important to proceed with ASCT. “Afraid of side effects” was the most common reason for declining ASCT. Fifteen oncologists completed the survey and 10 follow-up interviews were conducted. Most relied on the ‘eye-ball’ test for ASCT eligibility over geriatric screening tools. The lack of both high-quality evidence and local guidelines impacted decision-making. Both oncologists and patients felt that chronological age alone should not affect ASCT eligibility.

**Conclusion:**

While decision-making factors regarding ASCT can be variable, both oncologists and patients indicated that chronological age alone should not represent a barrier for ASCT among older adults. Future simplification and incorporation of ASCT eligibility geriatric assessment tools in studies as well as the inclusion of these tools in local guidelines may further improve ASCT decision-making.

## Introduction

Multiple myeloma (MM) is an incurable plasma cell neoplasm. It is a disease of older adults, with a median age of 70 years at diagnosis (1). Autologous stem cell transplantation (ASCT) remains the standard of care for eligible newly-diagnosed patients (2). Although many pivotal ASCT randomized controlled trials (RCT) excluded older adults (3, 4), several large retrospective studies and a meta-analysis have shown that ASCT is safe and feasible in selected older adults as per their local or study criteria (5, 6). There are a wide variety of tools that may be used to evaluate a patient for ASCT including performance status, geriatric assessment tools and frailty indices (7, 8); however, there a lack of agreement between these tools (9) and subsequently wide variation in their usage. Furthermore, there is wide heterogeneity in the eligibility of ASCT in older adults across different institutions and regions. To date, no studies have examined decision factors both from the perspective of patients and oncologists that influence ASCT eligibility specifically in older adults. Knowledge of these decisional preferences may lead to more tailored communication strategies enhancing shared patient-clinician encounters and ultimately optimizing ASCT usage among this patient cohort. To address this knowledge gap, we conducted a qualitative study to explore factors that influence decision-making regarding ASCT as a treatment modality among older adults with MM.

### Patients and methods

We conducted a qualitative study across two academic and two community centres in Ontario, Canada. Older english speaking adults with MM (newly diagnosed MM aged 65-75 in whom a decision had been made about ASCT in the previous 12 months) and treating oncologists were invited to participate in a baseline demographic survey and a subsequent interview between July 2019-July 2020. At each institution, ASCT is offered for eligible patients with MM who are not in progressive disease as per the International Myeloma Working Group criteria (10). All older adults with newly diagnosed MM aged 65-75 in whom a decision had been made about ASCT in the previous 12 months were eligible to participate. The upper limit of age 75 was used as ASCT was not offered at any of the participating sites above age 75. Patients were recruited between July 2019-July 2020 in clinics by study posters in the clinics, email mailers sent *via* local patient support group as well as *via* primary oncology teams. Information including baseline demographics was only collected on those participants who consented to the study. Interested participants reached out to the study coordinator either *via* their treating oncologist or *via* the email provided. The participants were subsequently screened for eligibility by the research coordinator and enrolled accordingly. Oncologists involved in the care of MM patients at the participating sites were contacted *via* email to fill out surveys. Semi-structured telephone interviews were conducted with patients and oncologists (see **Supplementary Table S1** and **Supplementary Table S2**). A thematic analysis was conducted to identify themes from the transcripts using qualitative analytical software (NVivo) (11). The initial 3 transcripts were independently read by two investigators (O.M and H.M) to develop a codebook. The codes were then applied to each interview subsequently and themes were developed. Patients were recruited until thematic data saturation was received for patients that underwent ASCT. Data saturation could not be reached for patients who declined ASCT. Patients who decline ASCT represent a minority of patients (12), and therefore were challenging to include in our study despite attempts aimed at specifically targeting them for recruitment. Given this uncommon clinical scenario, it was not feasible for us to continue to recruit patients until thematic saturation could be reached for patients that declined ASCT. Thisremains a limitation of our study and has been acknowledged in the discussion as well. The study was approved by the research ethics board of Hamilton Health Sciences and informed written consent was obtained from participants.

## Results

### Older adults with MM

**Figure 1:**
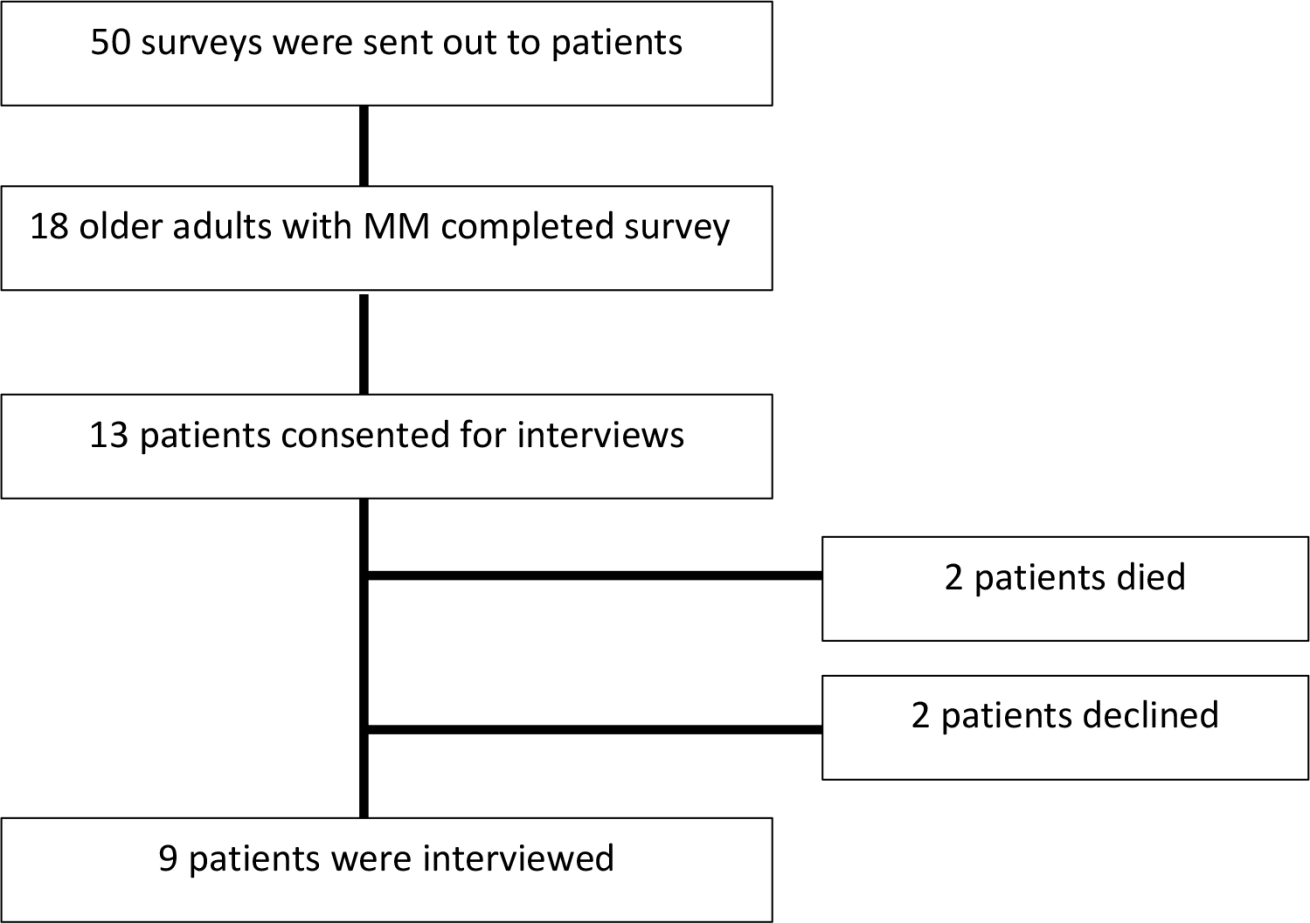
Flow chart showing those eligible patients vs participating patients.

**Figure 2:**
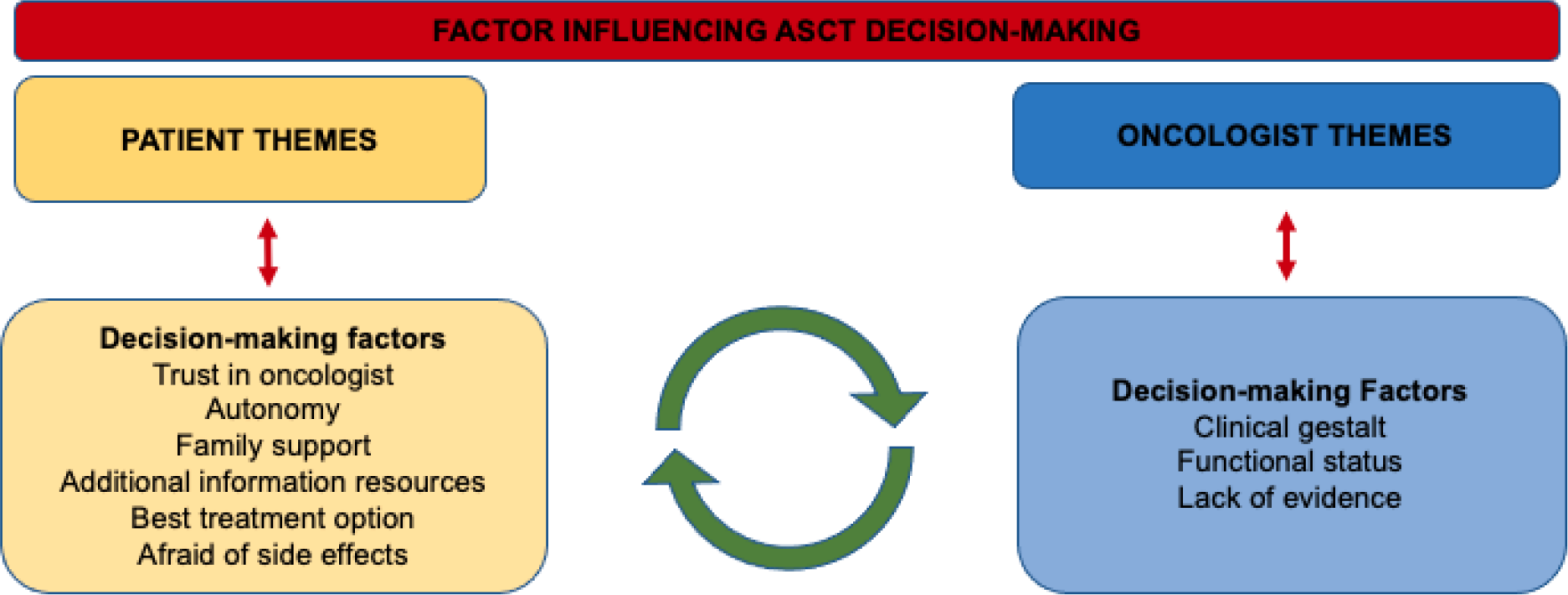
Common themes present in semi-structed interviews influencing autologous stem cell transplant decision-making among older adults with multiple myeloma.

A total of 18 older adults with MM completed the initial baseline survey (**Figure 1**). The median age of patients was 71 years and 50% (n=9) were female. A total of 78% (n=14) of patients were offered ASCT as a treatment option and, of those offered, 79% (n=11) proceeded with ASCT. Among these patients, 7/11 had completed the transplant within one year of the interview and 4/11 patients had a transplant scheduled in the upcoming six months. Additional patient details are outline in **Supplementary Table S3** in the supplement. Among the 18 patients that completed the baseline survey, a total of 9 follow-up interviews were conducted (13 patients consented for a follow-up interview; however, 2 patients died prior to interview date and 2 patients subsequently declined a follow-up interview). Only patients that were offered a transplant agreed to be interviewed. From the participants that agreed to be interviewed who were undergoing transplant, all the patients completed the interview after undergoing the transplant. The central themes related to decision-making are described in **Figure 2** with selected quotations in **Table 1**. “Importantly, in certain situations they were two oncologist involved in a patient’s care; a community oncologist that made the diagnosis and then an oncologist that specializes in transplant. Patients elucidated to both of these oncologist in the interviews.” Additionally, academic oncologists recommended patients for ASCT and community oncologist referred patients to be evaluated for ASCT.

**Table 1:** Selected quotes from each theme identified as influencing patient and oncologist decision making for an autologous stem cell transplant^*^.

Themes	Exemplar quotes
Older adults with MM	
Trust in my oncologist	*-”I have great trust in the knowledge of doctors”* *-”[oncologists] knowing what was best for me [the patient]”* *-”I have always trusted my doctor with my health and will always continue to do so.”*
Autonomy	*- “I trust my doctor’s recommendations, but I had the final say.”* *-”we had a one-hour meeting with the doctor, and then I decided to take their recommendation.”* *-”the final decision was all mine. I did ask my doctor for her recommendation, but the final decision was my own”* *-”I’m sure I could have said no I don’t want to do it.”*
My family	*-”we made our final decision that day after I discussed with my whole family.”* *-”we had a family meeting so they were involved they knew what was going to happen well in advance and my husband and I are extremely close so he was invaluable the whole time”* *-”and then I spoke to some of my family members to help make the decision.”* *-”there are a lot of emotional things you are dealing with, and most of the stuff goes over your head and you tend not to hear everything, so having a family member was great.”*
Other sources	*-”my doctor gave me a bunch of papers to read which I found very helpful to understand everything* *-”we would check things on the internet just to clarify certain things”* *-”My husband read it, I read portions of it but some of it got a little bit in depth. It’s important to read up and understand what you’re getting because it can make all the difference on your treatment.”* *-”My wife and I, we looked at the Internet constantly and read up on everything we could about Multiple Myeloma”*
Best option	*-”…emotional wellbeing was one of the factors in making the decision, but did not play a significant role as I [they] wanted the best possible treatment so I could function normally afterwards.”* *-”the decision I made was going to be the best option to put my disease into remission.”* *-”I was going to go with the best that was available”*
Afraid of side effects	*-”I was going to go for it, but then I just thought of having to stay in hospital for longer if I got more sick, and I didn’t want that.”* *-”I was already having such a hard time on the chemotherapy, and we just found this perfect dose, so didn’t want to touch it.”* *“I’m afraid of the infection and other side effects. you are worried about dying from the side effects”* *-”The main thing was I was scared of the side effects from the treatment.*”
Oncologists	
Clinical gestalt	*-”I do sort of an eye-ball, global test to determine eligibility.”* *-”What I would call the eyeball test where you just look at a person”* *-”Usually, I use an so called eye-ball test, or base the decision on my overall clinical gestalt.”* *-”when I walk into the room I’m pretty quick to make a decision about transplant eligible or not. And that is based on my gestalt”* *“when making a decision, I am absolutely looking at medical comorbidities, functional status, but most importantly, my clinical gestalt.”*
Functional status	*“if I feel the patient already has reduced functional status, I will shy away from transplant.”* *“they are so many tools, I never know which one to actually use.”* -”*If patient is not doing well functionally, that would very much deter me from doing the transplant”* *-”For the individual patient, I’m absolutely looking at functional status”*
Lack of evidence	*-”The biggest barrier is the lack of guidelines or evidence in this group of patients.”* *-”I wish there was more research that was conducted in this group of patients”* *-”I know different centres use different cut off’s for transplant”* *-”I wish there was more consistent ways to approach this”* *-”I think that [clear guidelines] would be very useful and I think a lot of the issue is that clinical trials are done in younger patients”* *“I can’t find any specific guidelines, so I try to look at whatever data there is myself.”*

*specific demographic for each participant are not described in order to decrease the risk of confidentiality loss.

#### Theme: “trust in my oncologist”

From the perspective of patients, factors that influenced their decision to proceed with ASCT included “*having a strong trusting and therapeutic relationship with [their] oncologist*”. Additionally, there were many patients who also felt “*the decision was made when the doctor said go with the transplant.*”

#### Theme: “autonomy”

Despite patients reporting their oncologists usually made the decisions for them, themes of patient autonomy were also present throughout the interviews. One patient reported, “*the final decision was all mine.*” In addition, most patients did mention going home to think about the doctor’s recommendation prior to making a decision.

#### Theme: “my family”

The inclusion of family in treatment decision-making surfaced in most interviews. One patient said, “*I found it helpful to discuss with my family before making a final decision.*” Additionally, patients also reported the significant benefit of having a family member come to their appointment.

#### Theme: “other sources to help decision making”

Patients also reported using pamphlets and brochures provided by their doctors as an additional resource in making a decision. Another patient reported that they “*read everything about it on the internet even though we are not supposed too*.”

#### Theme: “best option”

Patients reported that they ultimately wanted the “*best option for treatment*.” Another patient said, “*we just asked the doctor if this was the best option and if they said yes, we went with it*.”

#### Themes: “afraid of side effects”

Among the two included patients that declined ASCT, both stated that they were afraid of side effects and one commented that they were “*scared of being in hospital for prolonged period of time*.”

### Oncologists

**Figure 3:**
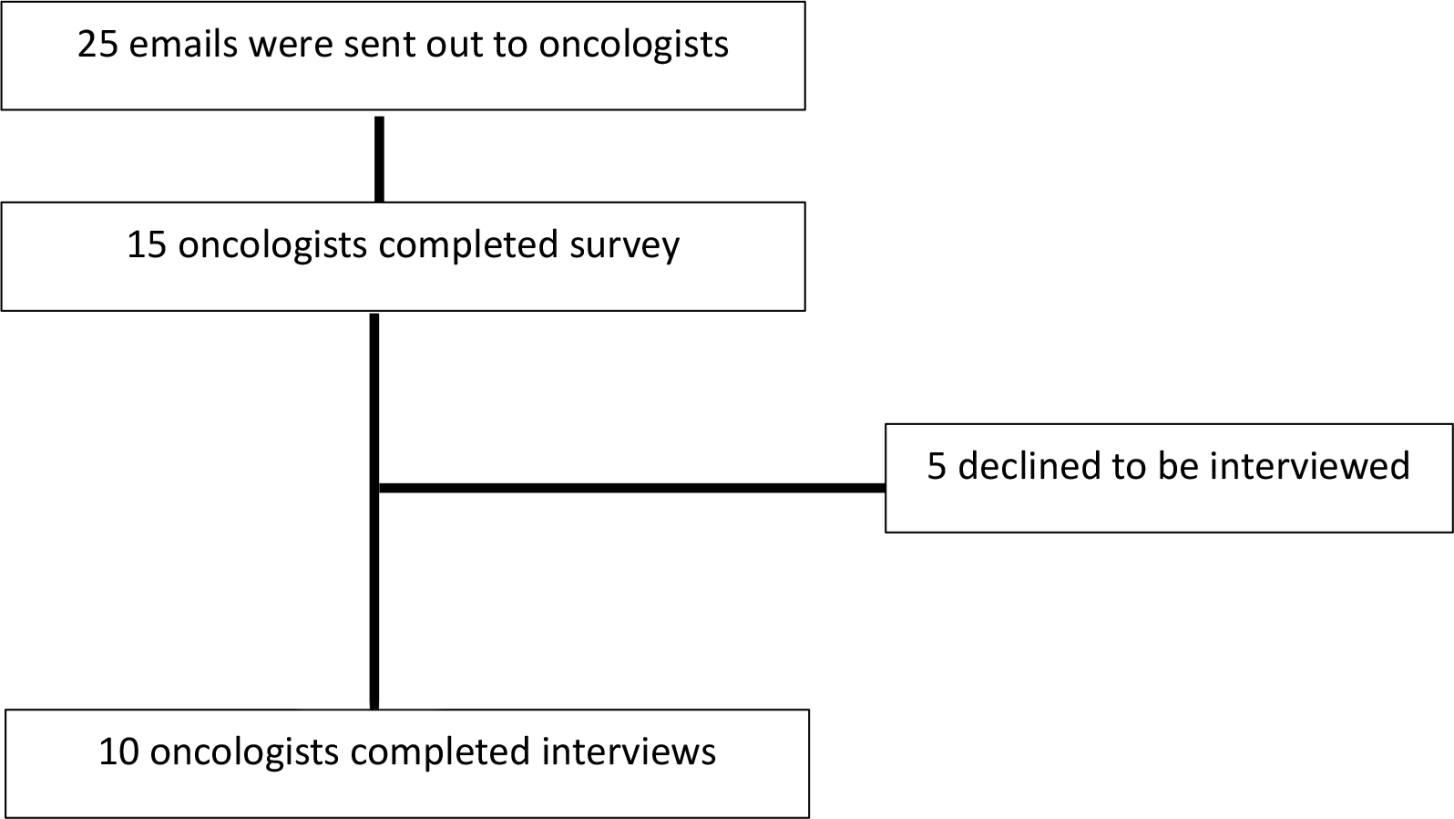
Flow chart showing those eligible oncologists vs participating oncologists.

A total of 15 oncologists completed the baseline survey (**Figure 3**). Most oncologists practiced at an academic centre (n=12, 80%) and the majority were in clinical practice for over 5 years (n=9, 60%). Additional oncologist details are outline in **Supplementary Table S4** in the supplement. Among the 15 oncologists that completed the survey, 10 oncologists agreed to a follow up interview and the central themes and selected quotations are illustrated in **Figure 1** and **Table 1** respectively. Additionally, community oncologists recommened patients to be evaluated for ASCT

#### Theme: “clinical gestalt”

A common theme was that although oncologists were aware of different geriatric assessment tools, many of them relied on the so called ‘eye-ball test’. One oncologist reported that “*as soon as I enter the room, I am very quick to either recommend transplant or not just by looking at the patient*.”

#### Theme: “functional status”

Oncologists reported that the patient’s functional status was important in recommending ASCT. When asked how physicians would assess the patient’s functional status, one reported *“I guess I could use one of those many different scales, but they take long.”* Most oncologists did mention that they would be in favor of a ‘targeted geriatric assessment tool’ to aid with ASCT decision-making.

#### Theme: “lack of evidence”

Oncologists reported that *“…the main barrier is lack of RCTs*” among older adults with MM that impact their decision-making regarding ASCT. Many oncologists felt that if they had clear and consistent local guidelines regarding ASCT, they would feel more comfortable with ASCT decision-making among older adults.

### Patient & oncologist perspectives: Impact of chronological age

Both physicians and patients felt that chronological age alone should not affect ASCT eligibility. One patient reported *“my age was not a factor at all in making this decision, it was more my gut feeling.”* Similarly, most oncologists stated that there should be no stringent chronological age criteria; however, when asked to recall patient cases, many had their own ‘personal’ age cut-offs. Some oncologists mentioned they would never transplant *“over age 72, and it would become a hard decision when they are in the 70-71 range.”*

## Discussion

Our study analyses the contextual factors from the perspective of oncologists and older adults with MM that specifically influences ASCT decision-making.

Our finding that most older patients accepted the treatment recommendation by their oncologist is consistent with previous studies (13, 14). Although most patients trusted their oncologist to make the decision, our study also showed that many older adults took a more active role in decision-making, often utilizing additional resources. As decisional preferences are known to vary among oncology patients (15), our study highlights the need to assess these preferences explicitly in order to tailor information and communication.

‘Best option’ for disease emerged as another significant theme which affected decision making. The definition of ‘best option’ likely varies for each individual patient and therefore treatment decision making needs to be guided by individual patient preferences and treatment goals (16). From the perspective of the oncologists, while most oncologists were aware of geriatric assessment tools, oncologists continued to rely on ‘clinical gestalt.’ Further studies specifically examining the role of geriatric assessment defining ASCT eligibility and utilization as well as their incorporation in guidelines is required to increase its usability.

The role of chronological age alone in the decision to offer ASCT was also interesting to note. Although most oncologists stated that chronological age alone did not impact their decision making, many had ‘age cut-offs’ which varied among clinicians even at the same treatment site. Chronological age alone is known to be a poor indicator of the physiological and functional status of older adults (17). The lack of RCT data was cited as the top barrier in recommending ASCT to older adults which emphasizes the need to ensure older adults are represented in trials (18).

The strengths of our study include a multi-perspective viewpoint from both oncologists and patients. There are several limitations of our study. We recruited patients at various stages and their recall may have been affected by the timing of the ASCT relative to the time of the interview. We also had limited participation for community centres. Additionally, we did not reach data saturation for patients who declined ASCT and therefore there may be additional factors which may have impacted their decision making that were not explored in our study and should be further studied. Our study was also conducted within a publicly funded provincial health care system in a relatively small geographic distribution; therefore, additional factors in health care settings as well as geographic constraints may result in different decision-making factors. Furthermore, with the availability of newer regimens for multiple myeloma especially in the transplant ineligible setting as daratumumab-lenalidomide-dexamethasone, it is possible that decision making factors regarding ASCT may have further changed over time. Also, our study excluded non-English speaking participants, which is also a limitation, as it may not represent the overall diverse Canadian population. Lastly, there is inherent bias in the patients and oncologists who agreed to both participate and subsequently be interviewed for this study and therefore both the demographics as well the factors which impacted decision-making cannot be determined for the participants who declined participation in this study.

In conclusion, our study demonstrates that while decision-making factors regarding ASCT can be variable both from the perspective of the patient and oncologist, both oncologists and patients feel that chronological age alone should not represent a barrier for ASCT among older adults with MM. Future incorporation of geriatric assessment tools in defining ASCT eligibility in studies as well as the inclusion of these tools in local guidelines may further improve decision making.

## Data availability statement

The original contributions presented in the study are included in the article/**Supplementary Material**. Further inquiries can be directed to the corresponding author.

## Ethics statement

The studies involving human participants were reviewed and approved by Research Ethics Board Hamilton Health Sciences. The patients/participants provided their written informed consent to participate in this study.

## Author contributions

Study concepts: OM, MP, AM, TW, MF, SA, HM. Study design: OM, MP, AM, TW, SA, HM. Data acquisition: OM, AM, MK, MS, HM. Quality control of data and algorithms: OM, HM. Data analysis and interpretation: OM, HM, TW, MP, MF, SA. Statistical analysis: OM, HM, MP, MF. Manuscript preparation: OM, HM: Manuscript editing/review: OM, MP, AM, TW, MF, MK, MS, SA, HM. All authors contributed to the article and approved the submitted version.
