# Supplementary material for: Decision-making factors for an autologous stem cell transplant for older adults with newly diagnosed multiple myeloma: A qualitative analysis
Source: Front Oncol. 2023 Jan 27;12:974038. doi: 10.3389/fonc.2022.974038 (PMC9911655; doi:10.3389/fonc.2022.974038)
Supplement: Supplementary file 1 [file DataSheet_1.docx]

**Supplementary Information**

**Table S1:** Semi-structured interview guide for older adults with MM

| Currently, where are you in your treatment for multiple myeloma?   1. Tell me a little bit more about the time when you were diagnosed with MM and what were some of the treatment options presented to you?    1. Did your doctor mention getting a transplant?    2. Were you satisfied with the treatment options that were presented to you?    3. How much information did you want to know regarding your disease?    4. How quickly did you make the decision about having transplant or not?    5. Did you feel the decision was made for you or you had the final say? 2. Overall, were you comfortable with how your oncologist communicated with you about your diagnosis and treatment options?    1. How would you like that kind of communication to be improved in the future?    2. Did you feel all your questions were answered? 3. Thinking back to when you made the decision for your treatment, how did you go about making that decision? What factors influenced your decision to accept/refuse the treatment? 4. Did any other factors contribute to your decision making, such as where you lived, your over-all well-being, your emotional and mental wellbeing? Other medical conditions? Anything else? 5. While you were making the decision about your treatment, did you consult with anyone else or look at any other resources? 6. When you were making the decision about your treatment, what was the most important benefit you hoped to achieve after the treatment option you choose? 7. While you were making the decision about your treatment, was there anything you were afraid of or worried about in choosing the treatment? 8. If you had to tell someone else who was undecided about undergoing a transplant, what would you tell them? 9. Lastly, how satisfied do you feel with the entire process from your diagnosis to management of your multiple myeloma? 10. Did you find any barriers or obstacles during the entire process that you experienced? Do you have any suggestions about how to improve the entire process of transplant? |
| --- |

**Table S2:** Semi-structured interview guide for oncologists

| 1. Tell me what do you know about the role of stem cell transplant in myeloma? 2. What are the benefits? What are the risks? 3. Do you always present transplant to older patients (age >65 years) with myeloma? 4. How do you decide who is transplant eligible? 5. Do you use any tools to make that decision? 6. Do you wish that there were tools or guidelines that better defined transplant eligibility? 7. Has your practice of transplanting older patients with myeloma changed over your clinical career? If so, how? 8. What are some reasons that you transplant more or fewer individuals with myeloma now than you used to? 9. How happy are you about your decision of transplant after you’ve made it? 10. If you don’t present the option of transplant to certain patients, what are some factors that influence your decision? 11. Where do you see the role of transplant in the next 5-10 years for older patients with myeloma? 12. What is your biggest worry about transplanting older patients? 13. Do you have any suggestions for improving in the transplant process for older patients? 14. Was there anything else you thought I would ask you today, but I didn’t? |
| --- |

**Table S3:** Baseline characteristics of newly-diagnosed older adults (age ≥65) with multiple myeloma

|  | **N=18** |
| --- | --- |
| Median age (range) | 71 (65-77) |
| Female sex (n, %) | 9 (50.0) |
| **Location of treatment (n, %)**  Academic  Community | 13 (72.0)  5 (28.0) |
| **Year of diagnosis (n, %)**  2017  2018  2019 | 1 (5.6)  7 (38.9)  10 (55.5) |
| **Co-morbidities (n, %)**  None  1-2  >2 | 6 (33.3)  9 (50.0)  3 (16.7) |
| **Total number of medications (n, %)**  <2  2-10  10+ | 5 (27.8)  12 (66.7)  1 (5.6) |
| **Functional Status (n,** %)  Fully independent  Loss of IADLs (≥1) | 9 (50.0)  9 (50.0) |
| **Treatment offered by Oncologist (n, %)**  ASCT therapy  *-Underwent ASCT**  *-Declined ASCT**  Non ASCT therapy | 14 (77.7)  *11 (78.6)*  *3 (21.4)*  4 (22.3) |

*(n, %) are for calculated among those where ASCT was offered by oncologist

ASCT autologous stem cell transplant; IADLs instrumental activities of daily living; MM multiple myeloma

**Table S4:** Baseline characteristics of treating oncologists

|  | **N=15** |
| --- | --- |
| **Location of practice (n, %)**  Academic  Community | 12 (80.0)  3 (20.0) |
| **Years in practice (n, %)**  < 5 years  5-10 years  >10 yeas | 6 (40.0)  2 (13.0)  7 (47.0) |
| **MM patients seen per week, both new and follow-up (n, %)**  <10  11-20  >20 | 8 (53.3)  4 (26.7)  3 (20.0) |
| **Percent of MM patients in clinic age ≥ 65 (n, %)**  0-25%  26-50%  51-75%  76-100% | 1 (6.7)  4 (26.7)  8 (53.3)  2 (13.3) |
| **Routinely referring MM patients (age 65-69) for ASCT (n, %)**  All the time/ most of the time  Some of the time/rarely | 15 (100)  0 (0) |
| **Routinely referring MM patients (age 70-75) for ASCT (n, %)**  All the time/ most of the time  Some of the time/ rarely | 3 (20)  12 (80) |
| **Oncologist Specialization**  General Oncologist  Multiple Myeloma Specialist  Transplant Physicians^#^ | 12 (80%)  3 (20%)  7 (46%) |

*All variables are as reported by oncologists #Transplant physicians could be either general oncologists or MM specialists; ASCT autologous stem cell transplant; MM multiple myeloma
